# Symmetrical arrangement of proteins under release-ready vesicles in presynaptic terminals

**DOI:** 10.1073/pnas.2024029118

**Published:** 2021-01-18

**Authors:** Abhijith Radhakrishnan, Xia Li, Kirill Grushin, Shyam S. Krishnakumar, Jun Liu, James E. Rothman

**Affiliations:** ^a^Department of Cell Biology, Yale University School of Medicine, New Haven, CT 06520;; ^b^Department of Microbial Pathogenesis, Yale University School of Medicine, New Haven, CT 06520;; ^c^Microbial Sciences Institute, Yale University School of Medicine, New Haven, CT 06520

**Keywords:** synaptic vesicles, cryoelecton tomography, SNARE protein, vesicle priming

## Abstract

Controlled release of neurotransmitters stored in synaptic vesicles (SVs) is a fundamental process that is central to all information processing in the brain. This relies on tight coupling of the SV fusion to action potential-evoked presynaptic Ca^2+^ influx. This Ca^2+^-evoked release occurs from a readily releasable pool (RRP) of SVs docked to the plasma membrane (PM). The protein components involved in initial SV docking/tethering and the subsequent priming reactions which make the SV release ready are known. Yet, the supramolecular architecture and sequence of molecular events underlying SV release are unclear. Here, we use cryoelectron tomography analysis in cultured hippocampal neurons to delineate the arrangement of the exocytosis machinery under docked SVs. Under native conditions, we find that vesicles are initially “tethered” to the PM by a variable number of protein densities (∼10 to 20 nm long) with no discernible organization. In contrast, we observe exactly six protein masses, each likely consisting of a single SNAREpin with its bound Synaptotagmins and Complexin, arranged symmetrically connecting the “primed” vesicles to the PM. Our data indicate that the fusion machinery is likely organized into a highly cooperative framework during the priming process which enables rapid SV fusion and neurotransmitter release following Ca^2+^ influx.

## Significance

Neuronal cells maintain a small number of synaptic vesicles (SVs) that are “primed,” i.e., ready to release upon receiving the triggering signal to allow for tightly regulated and rapid release of neurotransmitters. The proteins involved in this process are known, but how they assemble and operate together is poorly understood. Here we report the visualization of protein organization under primed SVs in cultured neurons under native conditions. Using cryoelectron tomography analysis, we find that there is a symmetric arrangement of exactly six protein densities at the docking interface, suggesting the fusion machinery is well ordered and prearranged for fast and precise release of neurotransmitters.

Neuronal communication is largely achieved by the controlled release of neurotransmitters stored in synaptic vesicles (SVs) into the synaptic junctions (1–4). The SV exocytosis involves a series of morphologically and molecularly defined sequential steps wherein vesicles first translocate to and are loosely “tethered/docked” to the active zone (AZ), then undergo preparatory molecular reactions termed “priming” which renders it fusion competent and culminates in membrane fusion, when triggered by an external stimuli (1, 4, 5). The extraordinary speed of synaptic transmission relies on a small subset of primed vesicles, the so-called readily releasable pool (RRP), that fuse within a few milliseconds upon Ca^2+^ influx (1–4). Despite the evident importance of such precision release, and despite all of the progress in our understanding of membrane fusion in general, we have no clear explanation for how it is achieved.

Vesicle fusion is catalyzed by the membrane-bridging complexes formed by the synaptic SNARE proteins, VAMP2 on the SV (v-SNARE) and Syntaxin1/SNAP25 (t-SNAREs) on the plasma membrane (PM) (6–8). Prior to fusion, the SVs are linked (or docked) to the AZ by tethering molecules, like RIM1α and Munc13-1 and subsequently converted into a state of fusion competence (1, 5). The current prevalent view is that on a primed RRP vesicle, the v- and t-SNAREs are engaged but the assembly process is arrested (clamped) in a “half-zippered” state (SNAREpins) (9–11). This prevents uninitiated spontaneous vesicular fusion events but allows for rapid Ca^2+^-evoked release (12, 13). The SNARE assembly and the resulting SV fusion is tightly regulated in the synapses by several proteins to enable Ca^2+^-regulated exocytosis (1, 4, 14). This includes molecular chaperones, Munc18-1/Munc13-1 which cooperatively catalyze efficient SNAREpin nucleation (4, 15), as well as the synergistic action of Complexin and presynaptic Ca^2+^-sensor Synaptotagmin-1(Syt1) to “clamp” SV release and synchronize the late SV fusion steps (4, 12, 13, 16).

In vitro reconstitution experiments show that the fusion of an isolated SV (or SV mimetics) with planar bilayers by the purified synaptic SNARE proteins takes 20 to 100 ms after the SNAREs engage to form a half-zippered (RRP-like) state (6, 17–19). While this is more than ample to explain membrane fusion in intracellular protein transport and endocytosis (20–22), the speed of SNAREs alone is 100 to 1,000 times too slow to explain synchronous neurotransmitter release. This discrepancy suggests that the SNARE proteins are specially organized in synapses, but exactly how remains poorly understood.

Cryoelectron tomography (cryo-ET) has emerged as a powerful tool in elucidating supramolecular structures both in vivo and in vitro (23–25). Particularly, cryo-ET imaging allows the cells to be captured as close as possible in their native state, while providing higher resolution compared to classical electron microscopy (EM) or optical microscopy techniques (26–28). Indeed, it has been successfully applied to reveal protein structures in cells at resolution sufficient to delineate their overall shape and locate protein constituents within (29–31).

Recently, cryo-ET analysis has been applied to investigate the arrangement of synaptic proteins under docked vesicles both in an in vitro reconstituted fusion system (32) and in neuroendocrine (PC12) cells (33). In the biochemically defined system, the docked SV mimetics were shown to have varying numbers of protein complexes with heterogeneous arrangements (32). The synaptic proteins formed “clusters” at the docking site at larger membrane separations but are organized into a “ring-like” arrangement when the membranes are closely apposed (32). In synaptic-like vesicles docked in neurite varicosities that develop from nerve growth factor (NGF)-differentiated PC12 cells (33), we observed six distinct masses, each corresponding to a single exocytic unit, arranged symmetrically at the SV–PM interface (33). Mutational analysis revealed that the symmetrical organization is likely templated by circular oligomers of Synaptotagmin (33).

These studies are a good approximation for RRP vesicles and provide valuable insights, but they have limitations. For example, the in vitro reconstitution system contained a majority, but not all of the critical protein components. In particular, it did not include Munc13-1 and employed preassembled t-SNARE complexes on the target membrane (32). The PC12 cells are a widely used model system to study neuroexocytosis but they contain a mixture of secretory vesicles, with a majority of large (∼100 to 150 nm) dense-core vesicles, and a small population of smaller, synaptic-like vesicles. Therefore, we used NGF differentiation to increase the proportion of synaptic-like vesicles and to induce neurite growth (33). Furthermore, NGF-differentiated PC12 cells lack mature active zones and do not maintain a pool of release-ready vesicles. Thus, we overexpressed a VAMP2 protein with mutations in the C-terminal half (termed VAMP2-4X) which allows SNAREs to zipper approximately halfway but not completely, and as such allows vesicles to dock but not fuse (33, 34). Additionally, due to the orientation of the PC12 cells relative to the electron beam, the plasma membrane was not visible in the subtomograms (i.e., missing wedge effect), and its position was only inferred indirectly (33).

Hence, we sought to visualize the organization of the exocytic machinery on docked SVs, particularly under the primed vesicle, in presynaptic terminals under native conditions. Here, we describe results from a cryo-ET analysis of SVs at different stages of docking in cultured hippocampal neurons. Our imaging analyses indicate that protein arrangement on a SV is dependent on or a function of the intermembrane distances and there is a symmetric arrangement of exactly six protein densities, each possibly corresponding to a single SNARE-associated exocytic module, at the SV–PM interface of the primed RRP vesicles.

## Results

**Fig. 1.**
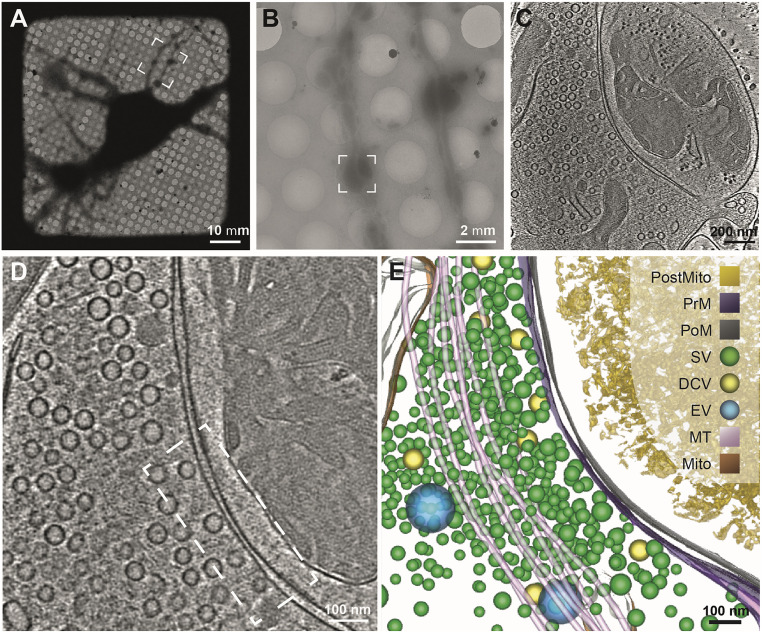
Cryoelectron tomography analysis of synaptic vesicles in primary hippocampal neurons. (*A*) Low magnification (220×) cryo-EM micrograph of the hippocampal neurons cultured directly on Au grids revealed the presence of neuronal networks of varying densities. (*B*) Cryo-EM micrograph at 3,600× magnification of the highlighted area (dashed white box in *A*) revealed the presence of neuronal boutons. (*C*) High magnification cryo-EM micrograph of the synaptic bouton (highlighted by the dashed white box in *B*) was collected at 26,000× using Titan Krios equipped with a Volta phase plate and energy filter. (*D*) Representative tomographic slice and corresponding 3D segmentation rendering (*E*) shows the distribution of synaptic vesicles in the neuronal boutons. Both the tomographic slice and the 3D segmentation revealed the presence of synaptic vesicles (SVs) at different stages of docking and the neuronal ultrastructure such presynaptic membrane (PrM), postsynaptic membrane (PoM), dense core vesicles (DCVs), endosomal vesicles (EVs), microtubules (MTs), mitochondria (Mito), and postsynaptic mitochondria (PostMito).

To investigate the molecular architecture under the docked SVs, we employed cryoelectron tomography analysis on hippocampal neurons in culture. The experimental workflow is outlined in *SI Appendix*, Fig. S1. Briefly, primary neurons derived from P0 mice were cultured directly on sterile Quantifoil R2/1 -gold (Au) grids and grown for 14 to 17 days in vitro (DIV14 to 17) prior to being plunge frozen into liquid ethane cooled down by liquid nitrogen. The neuronal varicosities were thin enough allowing the formation of vitreous ice during flash freezing. This enabled direct imaging of the well-preserved neuronal synapses at their native form using cryo-ET without using any thinning procedures as evidenced by various membranous and cytoskeletal structures (Fig. 1). The frozen-hydrated specimens were imaged using a 300 kV Titan Krios transmission electron microscope equipped with a Volta phase plate (VPP) and an energy filter mounted in front of a K2 Summit direct electron detector.

We first obtained full-montage at 220× magnification in low dose mode to create a map of the intricate neuronal network in the frozen grids. The map was used to locate grid squares with neurites (or axons) that are thin enough for imaging using cryo-ET (Fig. 1*A*). We then collected a series of small montages at 3,600× magnification along the selected thin extended neurites to identify regions with axonal varicosities with closely apposed membranes resembling neuronal synapses (Fig. 1*B*). Tilt series images were acquired at 26,000× magnification at these selected sites (Fig. 1*C*). The image stacks were subjected to motion correction and aligned using the fiducial gold markers. The aligned tilt series was then reconstructed to generate a three-dimensional (3D) tomogram. A representative tomographic slice and the resultant 3D segmentation are shown in Fig.1 *D* and *E* and Movies S1, S2, and S3.

The tomograms revealed characteristic features of a neuronal synapse, with the plasma membrane of the presynaptic terminal (containing lots of SVs) in close apposition with the membrane of the target (postsynaptic) site. We observed a large number of SVs within the presynaptic terminal, locating at varying distances from the presynaptic membrane (PrM). To identify the docked SVs, we focused on vesicles that were located in close proximity (<20-nm interbilayer distance) to the PrM.

We observed 7,527 putative docked SVs from the ∼300 tomograms of primary neurons. A total of 2,556 of them were in a “side view” wherein the PrM was distinctly visible. In the remainder (4,971) subtomograms, the target membrane was not visible due to the missing wedge effect. In these cases, we adapted the protocol we developed previously (33) and used the XZ slice of the tomographic 3D reconstruction to initially screen and identify PM-proximal vesicles. We subsequently carried out Z-stack analysis on the XY slices and the absence of any organelles between the periphery of the vesicle and the boundary of the cell was used as the criteria to define docked vesicles. These were termed “top/bottom view.”

**Fig. 2.**
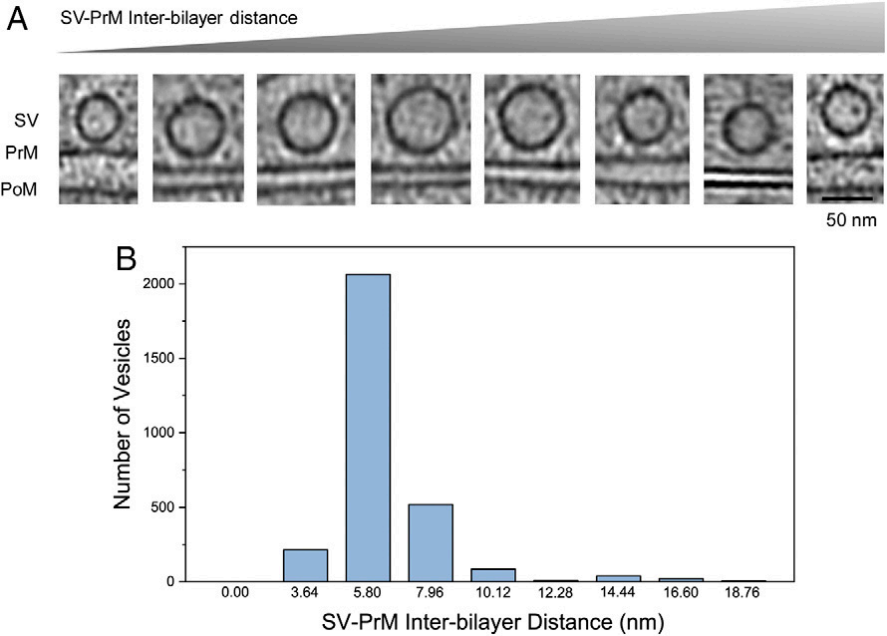
Classification of side-view docked vesicles as a function of interbilayer distance. (*A*) Representative tomographic slices of SVs at various stages of docking and (*B*) quantification of the observed distribution of docked synaptic vesicle (SV) based on the SV to presynaptic membrane interbilayer distance (*Bottom*) are shown. Side-view vesicles (∼2,500 bin4 subtomograms with 1 pixel = 2.16 nm) were used for the analysis.

For the side-view vesicles, we observed SVs at various stages of docking (Fig. 2A and Movie S2). So, we systematically measured the interbilayer distance and classified the vesicles as a function of the SV–PrM interbilayer distance (Fig. 2*B*). A majority of the side-view vesicles (∼75%) were found to be intimately docked to the PrM, with interbilayer distance ≤6 nm. In each instance, we detected strong protein density at the center connecting the SV to PrM (Fig. 2*A*). These vesicles were morphologically and structurally similar to the primed RRP vesicles (35–37). The rest of the side-view docked SVs (∼25%) located at differing distances from the PrM, with the interbilayer distance ranging from ∼8 to 20 nm (Fig. 2*A*). These vesicles were tethered to the PrM by a range of protein densities with no apparent order.

To obtain structural insights into the protein organization at the docking site, we grouped them into two separate pools—those with interbilayer distance ≤6 nm (termed primed) and those with interbilayer distance ≥8 nm (termed tethered) and subjected to several rounds of classification and alignments. The subtomograms within each group were first aligned with the vesicle and PrM and then subjected to further alignment and classification based on features at the SV–PrM interface. After multiple cycles of alignment and classification, the subtomograms of vesicles in each group were averaged together, both with and without sixfold rotational symmetry.

**Fig. 3.**
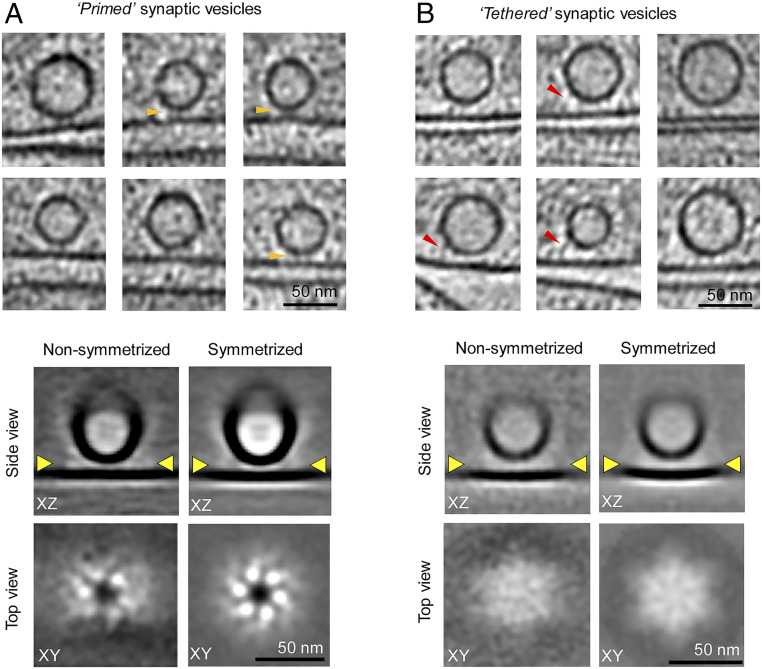
Cryo-ET 3D reconstruction of protein organization under docked vesicles. (*A*) Representative tomographic slices of primed SVs that have a SV–PrM interbilayer distance of ≤6 nm (*Top*). Pronounced protein density at the docking site is marked by orange arrowheads. The 3D reconstruction of the observed protein density, following several rounds of alignment and classification, revealed a hexameric arrangement of protein densities at the docking interface either with or without a sixfold rotational symmetry imposed (*Bottom*). (*B*) Representative tomographic slices of the tethered SVs that have a SV–PrM interbilayer distance of ≥8 nm (*Top*). These SVs were connected to PrM by varying numbers of long protein tethers (red arrowheads), but averaging failed to reveal any organized structure at the SV–PrM docking interface (*Bottom*). The yellow arrowheads represent the vertical position in the SV–PrM interface (in the XZ slice or side view) at which the XY plane (or top view) is rendered.

In the resultant 3D reconstruction of the primed pool (i.e., interbilayer distance ≤6 nm), we observed six rod-like densities in the XY plane corresponding to the vesicle–cell membrane interface (Fig. 3*A*). These protein densities, each connecting the SV to the PrM, were symmetrically organized in both symmetrized and nonsymmetrized reconstructions (Fig. 3*A*). In contrast, subtomogram averaging did not show any organized structure for the tethered pool of vesicles with interbilayer distance of ≥8 nm, although there were protein tethers connecting the SV to the PrM in individual subtomograms (Fig. 3*B*). Together, these data suggest that the exocytic protein machinery becomes highly organized when the vesicles are primed to release.

We used a strategy that we developed previously to align and classify the top/bottom-view vesicles (33). These vesicles were first sorted based on their diameter (*SI Appendix*, Fig. S2) and the 3D classes featuring spherical vesicles with diameters of 43.88 ± 5.32 nm were then selected to generate a homogeneous dataset. These were further subjected to several cycles of alignment and global averaging (using the vesicle center as reference) until there was no further improvement in the homogeneity in size and shape. The remaining vesicles (∼3,000 in total) were pooled together for local alignment and averaging with the center of alignment now at the site of docking. After several cycles of alignment and classification, the subtomograms were averaged together, both with and without sixfold rotational symmetry. The resulting density map (for both symmetrized and nonsymmetrized reconstructions) showed six pronounced protein densities symmetrically organized at the site of docking (*SI Appendix*, Fig. S2), nearly identical to that observed with the primed side-view vesicles. Interestingly, we observed faint density corresponding to the PrM in the averaged density map, even though it was not visible in the individual subtomograms. We believe that this faint density corresponding to PrM is likely due to contamination of the top/bottom-view dataset with a small number of the side-view particles.

**Fig. 4.**
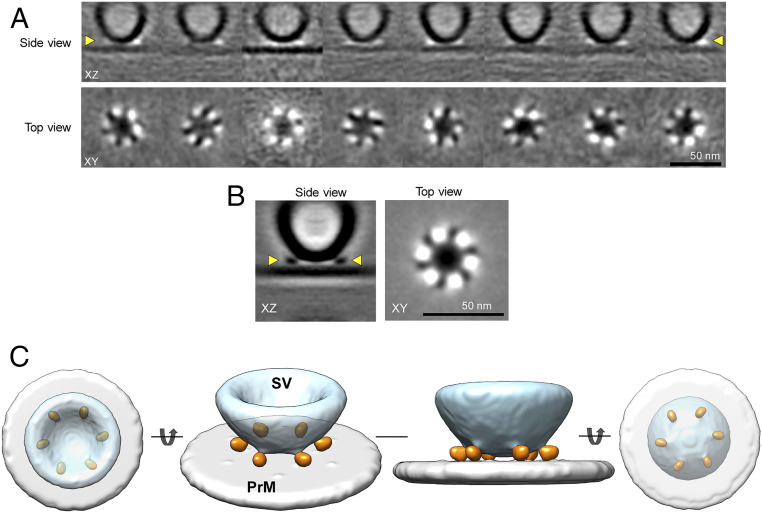
Symmetrical arrangement of protein density under docked synaptic vesicles. (*A*) Subtomogram class averages of SVs that are docked to the PrM. All the eight 3D class averages revealed a symmetrical organization of proteins at the SV–PrM interface. Side-view vesicles, with SV–PrM interbilayer distance of less than ∼6 nm and top/bottom-view vesicles, with a diameter of 43.88 ± 5.32 nm were pooled together for the subtomogram analysis. *Top* row: Slice through the center of the tomogram along the *z* axis. *Bottom* row: Corresponding slices through the volume in the XY plane at the vertical position highlighted by yellow arrowheads on the *Top*. (*B*) The cryo-ET 3D reconstruction of the SV–PrM interface reveals a symmetrical organization of six protein densities at the SV–PrM interface. (*Left*) Slice through the reconstructed volume along the *z* axis of the subtomogram averaged structure (XZ slice, side view) revealed protein densities connecting the SV to the PrM. (*Right*) Slice through the volume in XY plane (top view) at the vertical position highlighted by yellow arrowheads in the XZ slice revealed the symmetrical arrangement of protein densities at the SV–PrM interface. (*C*) Surface representation of the cryo-ET 3D reconstruction of the protein organization at the SV–PrM interface at different orientations. The cryo-ET 3D map at threshold level σ = 0.85 was segmented using UCSF Chimera for the surface representation. The top, side, and bottom views of the 3D reconstructions reveal six symmetrically organized protein densities (orange) connecting the SV (light blue) to the PrM (light gray).

To get a higher resolution of the structural elements at the SV–PrM interface, we pooled together subtomograms of all the side-view and top/bottom-view vesicles that yield a symmetrical arrangement of protein densities. After several rounds of alignment and classification (with site of docking as a reference), the tomograms were sorted into eight 3D classes (Fig. 4*A*). Remarkably, in all 3D classes we observed six densities in the XY plane corresponding to the SV–PrM interface (Fig. 4*A*). To further improve the resolution, all subtomograms in each of the eight 3D classes were pooled together and averaged with imposing sixfold rotational (*C*_*6*_) symmetry (Fig. 4*B*). The resulting density map showed six pronounced protein densities at the SV docking interface that were symmetrically arranged circumscribing a circle of ∼38-nm diameter. The surface representation of the resulting 3D map (at ∼4.4-nm resolution) revealed that the SV and the presynaptic membrane are approximately ∼3.5 nm apart and each of the six radially organized protein masses connect the SV to the PrM (Fig. 4*C*). Considering the strong signal, we reason that each of these densities likely correspond to a highly structured and large protein complex (*Discussion* and *SI Appendix*, Fig. S3). The noted protein organization was unique and specific to the docking interface and we observed no strong electron density on the vesicle outside the PrM contact regions.

## Discussion

Here we describe an in situ molecular imaging of protein structures on docked SVs under native conditions in hippocampal neurons. We find that SVs are initially connected to the PrM by ∼8- to 18-nm-long protein densities of variable number and heterogeneous arrangement. These tethering densities were observed only in the individual subtomograms, but not in the subtomogram class averages. It is possible that densities are disorganized and get smeared out during the averaging process. It is also worth noting that any limited organization might be missed due to the relatively small number of vesicles in the tethered pool. Based on the available structural and functional data, we believe that these tethering densities likely correspond to known SV tethering factors, like RIM1α and Munc13 (1, 14, 38) but cannot be conclusive at the present resolution.

The SVs progress to the primed state wherein they are uniformly held ∼4 nm away from the PrM by a symmetrically arranged protein complex. In this state, the synaptic SNARE proteins (VAMP2 in SV; Syntaxin/SNAP25 on PrM) are expected to engage and are clamped in a partially zippered prefusion state by regulatory factors, namely Syt1 and Complexin (12, 39, 40). Recent biochemical and structural data indicate that each “exocytosis module” likely involves a dual-clamp arrangement with a SNAREpin held in a vice-like grip by two Syt1 C2B domains, one independently (primary interface) and other in conjunction with Complexin (tripartite interface) (39, 40). We speculate that each of six protein masses we have discovered consists of one such module. Indeed, modeling shows that shape/size of each density can accommodate a single exocytosis module (*SI Appendix*, Fig. S3). At the present resolution, it is not possible to assign specific proteins and their actual orientation to the observed densities. However, it is tempting to speculate that exactly six SNAREpins are organized under the primed vesicles and they act cooperatively to drive ultra-fast SV fusion and rapid release of neurotransmitters.

What orchestrates the symmetrical (or hexameric) organization of protein densities at the SV–PrM interface? In the present structure, we could not resolve any protein densities connecting the six symmetrically placed units. But the symmetrical positioning along a circle of ∼38-nm outer diameter suggests that there is an underlying framework templating the observed arrangement. We have recently demonstrated that the Syt1 can polymerize into ∼30- to 40-nm diameter-sized ring-like oligomers and these oligomeric structures are critical for Syt1 function as a fusion clamp (41–44). We thus postulate that each observed density likely corresponds to a single SNAREpin bound to a Syt1 ring oligomer (via the primary interface) at regular intervals (12, 33). In support of this, we have previously demonstrated that destabilizing the Syt1 ring-like oligomers disrupts the organized structure under docked vesicles in NGF-treated PC12 cells (33). It is worth noting that at the present resolution, the Syt1-ring oligomer might not be visible due to its small thickness (∼4 nm) and high density/contrast of the presynaptic membrane.

Furthermore, VAMP2 has been shown to be preorganized within the SV into hexameric units by interactions with multispanning SV membrane protein synaptophysin (45, 46). In fact, among the proteins known to participate in the SV exocytosis, Synaptophysin is the only protein that exhibits a sixfold symmetry (45, 46). We thus, speculate that the VAMP2/Synaptophysin hexamer and Syt1 oligomers play a synergistic role in templating the symmetrical arrangement of six exocytic modules at the SV–PrM interface.

How precisely six SNAREpins are assembled under each SV, especially since there is an excess of both v- and t-SNAREs locally, remains unclear. We have previously hypothesized that SNARE-associated chaperone, Munc13-1, which orchestrates the assembly of the ternary SNARE complex, might play a crucial role in this process (12). Based on known structural features, we noted that six curved Munc13 MUN domains can surround an inner Syt1-ring and such a “buttressed ring” organization will naturally template precisely six SNAREpins, sterically block their full assembly, and cooperatively release them upon Ca^2+^ influx (12).

Our data suggest that six SNAREpins are maximally involved in catalyzing neurotransmitter release. While this might be surprising, however, there is emerging evidence that—if synchronously released—a handful of SNARE complexes could achieve a submillisecond fusion pore opening. Using in vitro bulk fusion analyses, it has been established that a single SNARE complex can drive lipid mixing and three SNAREs are sufficient to open a stable fusion pore (47, 48). Recent single-vesicle reconstitution experiments show that a relatively small number (∼10 to 12 copies) of SNAREs and Syt1 are necessary and sufficient to achieve rapid (∼100 ms) and Ca^2+^-synchronized vesicular fusion (6, 18, 19). In fact, recent modeling studies considering the concept of mechanical coupling have predicted that an optimum of four to six SNAREpins is required to achieve submillisecond vesicular release (49). Notably, physiological studies show that two to three SNARE complexes can be sufficient to facilitate Ca^2+^-evoked synchronous neurotransmitter release (50, 51). In summary, our data provide compelling evidence that a small number of SNAREpins prearranged into a cooperative structure represents a central organizing principle of Ca^2+^-regulated exocytosis.

## Materials and Methods

The overall workflow of experimental procedures is illustrated in *SI Appendix*, Fig. S1.

### Primary Culture of Hippocampal Neurons on EM Grids.

Primary mice hippocampal neurons were cultured as described previously (52). In brief, cells were isolated from P0 C57/B6 mice hippocampi. Hippocampi were dissected, treated with 0.125% trypsin, and dissociated into single cells by gentle trituration in Hanks’ balanced salt solution (HBSS) containing 10% fetal bovine serum (FBS), 10% bovine serum albumin (BSA), Glutamax and DNase. Next, cells were resuspended in Dulbecco’s modified Eagle medium (DMEM)/F12 containing 10% FBS, and then seeded at a density of 75,000 cells/mL on EM grids.

Specifically, we used Quantifoil R2/1 Au grids (200 mesh with holey carbon film of 2-µm hole size and 1-µm spacing, Electron Microscopy Sciences) and grids were prepared prior to seeding as follows: Grids were placed at the center of a 35-mm glass bottom MatTek dish (0.15 mm in thickness and 2 mm in diameter; MatTek Corp.). The carbon side of the grids were glow discharged for 25 s at 15 mA in a plasma cleaner (PELCO easiGlow, Ted Pella). After extensive washing with distilled water, the grids were sterilized in 70% ethanol for 10 min under ultraviolet (UV) light and then coated with 0.1 mg/mL poly-D-lysine (Thermo Fisher Scientific) overnight in a 37 °C incubator. The grids were subsequently washed with distilled water and incubated with HBSS and Neurobasal (NB) (Thermo Fisher Scientific) media for at least 12 h each before seeding the cells. Cultures were maintained in a humidified incubator for 14 to 16 d with 5% CO_2_ at 37 °C and the medium was changed every 3 d. Appropriate care was taken during the washes and medium exchange steps to avoid touching the grids and to prevent them from drying out. All animal care procedures were performed by strictly following the guidelines approved by Yale University Institutional Animal Care and Use Committee.

### Cell Vitrification.

After checking the neuron confluence and grid integrity under the light microscope at DIV14 to 17, BSA-coated 10-nm Gold Tracer beads (Aurion) were applied to the grids as fiducial markers, immediately prior to vitrification. Grids were blotted from the back side for 4 s using Whatman #1 filter paper (Sigma-Aldrich) and rapidly plunge frozen into liquid ethane cooled down by liquid nitrogen using a homemade gravity-driven plunger apparatus.

### Cryoelectron Tomography.

The cryo-ET imaging of the frozen hydrated samples at −170 °C was performed on a 300-kV FEI Titan Krios microscope (Thermo Fisher Scientific) equipped with a VPP and an energy filter fitted in front of a K2 Summit direct electron detector (Gatan) operated using SerialEM (version 3) software (53). Initially, a 9 × 10 full montage was recorded at low magnification (220×) to produce a complete grid overview. After identifying neuronal varicosities in the 220× montage, 2 × 2 montages were acquired at 3,600× to identify regions with thin enough ice suitable for cryo-ET imaging. A total of 296 tomographic series were acquired with the following parameters: 26,000× magnification; tilt range ± 51°; tilt increment 3°; total dose ∼50 e^−^/Å^2^; pixel size 5.4 Å. The K2 camera was operated in dose fractionation mode to generate 8 to 10 frames per projection image. The real-time phase shift was calculated with GCTF software (version 1.06), and changed to the next VPP when the phase shift was over 135^°^.

### Tomogram Reconstruction.

The dose fractionated projection images were first subjected to motion correction using MOTIONCOR2 (54) and then assembled into drift-corrected stack files by TOMOAUTO (55). These were subsequently aligned using gold fiducial markers or fiducial free cross-correlation using the IMOD (56, 57) software package. Tomograms were reconstructed from the aligned stacks by the simultaneous iterative reconstruction technique (58) (SIRT). The tomograms were further binned two and four times (hereafter called bin2 and bin4 tomograms) resulting in pixel sizes of 10.8 Å and 21.6 Å. BSA-coated fiducial gold beads were utilized to define the top membrane. Organelle structures were manually segmented using IMOD (56).

### Identifying Docked Synaptic Vesicles.

To identify the docked SVs, we focused on vesicles that were located close to the PrM. There were three types of docked SVs. The SVs that were directly connected to the PrM via protein tethers were manually picked and termed as side view. In this case, the PrM was distinctly visible in the 3D tomogram. There were also SVs that were located close/proximal to the top or bottom PM. But, the top/bottom PMs were not visible in the tomogram due to the missing wedge effect. We used the XZ slice of the tomographic 3D reconstruction to initially screen and identify PM-proximal vesicles. For this subset of vesicles, we further carried out Z-stack analysis on the XY slices and the absence of any organelles between the periphery of the vesicle and the boundary of the cell was used as the criteria to define docked vesicles. These were termed top/bottom view.

### Subtomogram Analysis.

A total of 7,527 synaptic vesicles were manually picked and extracted from 296 tomograms for subtomogram averaging using the I3 (0.9.9.3) software package (59, 60). Bin4 tomograms were used for particle picking since they provided higher contrast. The initial subtomogram positions and orientations were defined by the coordinates of the center of the SV and the coordinates of the position in the PrM closest to the SV. Subtomograms (256 × 256 × 256) of each SV were extracted from the original tomograms. Conventional imaging analysis, including 4 × 4 × 4 binning and low-pass filtering, were used to enhance the contrast of the subtomograms.

After an initial alignment based on the global average, multivariate statistical analysis and hierarchical ascendant classification were applied to sort vesicles with varying geometry and sizes. The bin4 subtomograms of the docked SVs were first sorted into 200 3D classes by the “alignment by classification” method (60). The 200 3D class averages were split into two groups based on the presence or absence of the PrM. The 3D class averages which exhibited both SV and PrM were pooled together as the side-view vesicles. The remaining classes with SVs that did not have a visible PM were pooled together and classified as the top/bottom-view vesicles. Out of the 7,527 subtomograms generated, 2,556 belonged to the side view and 4,971 belonged to the top/bottom view.

For the side-view SVs, the closest distance between the centers of the SV membrane to the centers of the PrM was measured using ImageJ software (version 1.52d, NIH). We subtracted 5 nm (average thickness of a lipid bilayer) from the center-to-center distance to get the SV–PrM interbilayer distance. A two-dimensional (2D) plot was generated with number of SVs in the *y* axis and SV–PrM interbilayer distance in the *x* axis (Fig. 2). Side-view vesicles with SV–PrM interbilayer distance of ≤6 nm or ≥8 nm were pooled together separately for further alignment and averaging.

The top/bottom-view vesicles were sorted based on their diameter. We first measured the distance between the centers of the bilayers at diametrically opposite ends of the vesicle for all the 3D class averages which belonged to the top/bottom view using ImageJ software (version 1.52d). The vesicles were assumed to be spheres for calculation. A total of 5 nm (average thickness of a lipid bilayer) was added to the center-to-center distance to calculate the diameter of the SV. A 2D plot was generated with number of SVs in the *y* axis and diameter of the vesicle in the *x* axis (*SI Appendix*, Fig. S2). The 3D classes featuring spherical vesicles with diameters of 43.88 ± 5.32 nm were then selected to generate a homogeneous dataset.

IMOD (56) was used to generate TIFF files required for the vesicle diameter and SV–PrM interbilayer distance measurements. Vesicle diameters and SV–PrM interbilayer distances (in pixels and nanometers) were measured using ImageJ software (version 1.52d) and Excel and Origin [Origin(Pro), version 2019, OriginLab Corporation] were used to carry out the relative frequency statistical analysis.

These selected subtomograms were combined and initially aligned using the vesicle and membrane (large mask). Once the subtomograms were aligned well with the SV and PrM, further alignment and classification were carried out using masks focused on the features at the SV–PrM interface. Subsequently, we used bin2 subtomograms (128 × 128 × 128 voxels) for further refinement. We explored the possibility of asymmetry (*C*_*1*_), but the resolution remained low. Higher resolution in situ structures were determined when *C*_*6*_ symmetry was imposed. The resolution of the final 3D reconstruction was estimated using EMAN2 (61) (*SI Appendix*, Fig. S4). The cryo-EM density map has been deposited in the Electron Microscopy (EM) Data Bank under accession number EMD-23077.

### Modeling and Visualization.

University of California, San Francisco (UCSF) Chimera (62) was used to visualize the tomogram and subtomogram structures in 3D. All the density maps were segmented using UCSF Chimera (62). UCSF Chimera (62) and ChimeraX (63) were used for surface rendering and visualization of cryo-ET maps and models. The X-ray crystal structure 5W5C (SNARE-Syt1-Complexin) was manually fitted into the cryo-ET map using USCF Chimera (62).

## Supplementary Material

Supplementary File

Supplementary File

Supplementary File

Supplementary File

## Data Availability

All study data are included in the article and/or supporting information.
