# Supplementary material for: Symmetrical arrangement of proteins under release-ready vesicles in presynaptic terminals
Source: Proc Natl Acad Sci U S A. 2021 Jan 18;118(5):e2024029118. doi: 10.1073/pnas.2024029118 (PMC7865176; doi:10.1073/pnas.2024029118)
Supplement: Supplementary File [file pnas.2024029118.sapp.pdf]

Supporting Information

Symmetrical Arrangement of Proteins under Release-Ready Vesicles in Pre-Synaptic Terminals

Abhijith Radhakrishnan[1†], Xia Li[2, 3, †$], Kirill Grushin[1], Shyam S. Krishnakumar[1*], Jun Liu[2, 3, *], James E. Rothman[1*]

[1]Department of Cell Biology, [2]Department of Microbial Pathogenesis, [3]Microbial Sciences Institute, Yale University School of Medicine, New Haven, CT 06520, USA.

[$]Current address: Institute of Special Environmental Medicine, Co-innovation Center of Neuroregeneration, Nantong University, Nantong, Jiangsu 226001, China

[†]These authors contributed equally

*To whom correspondence maybe addressed.

Email: shyam.krishnakumar@yale.edu, jliu@yale.edu; james.rothman@yale.edu

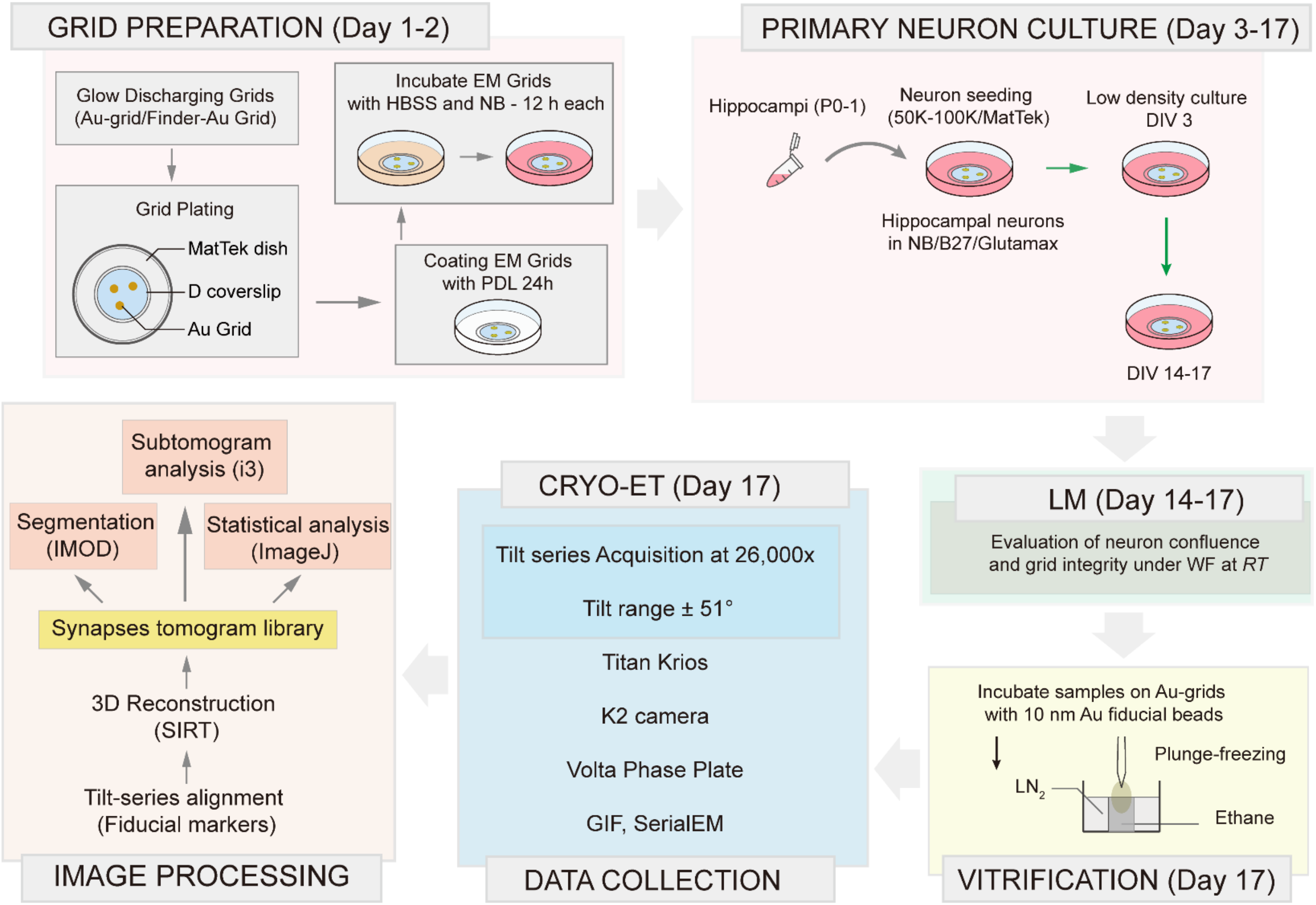

Fig. S1*: Work-flow for Cryo-ET Imaging of primary culture of hippocampal neurons.*

Primary neurons isolated from mice hippocampi were cultured directly on EM grids for 14-17 DIV. The EM grids were evaluated using light microscope for neuron confluence and were rapidly plunge-frozen into liquid ethane cooled down by liquid nitrogen using a homemade plunger. The frozen grids were imaged on Titan Krios to obtain a full-montage recorded at 220× using SerialEM software to map the neuronal network. Intermediate magnification montages at 3,600× were obtained to select neuronal varicosities best suited for tilt series data collection at 26,000×. The dataset was then reconstructed and further processed by segmentation, measurement analysis, and sub-tomogram averaging analysis.

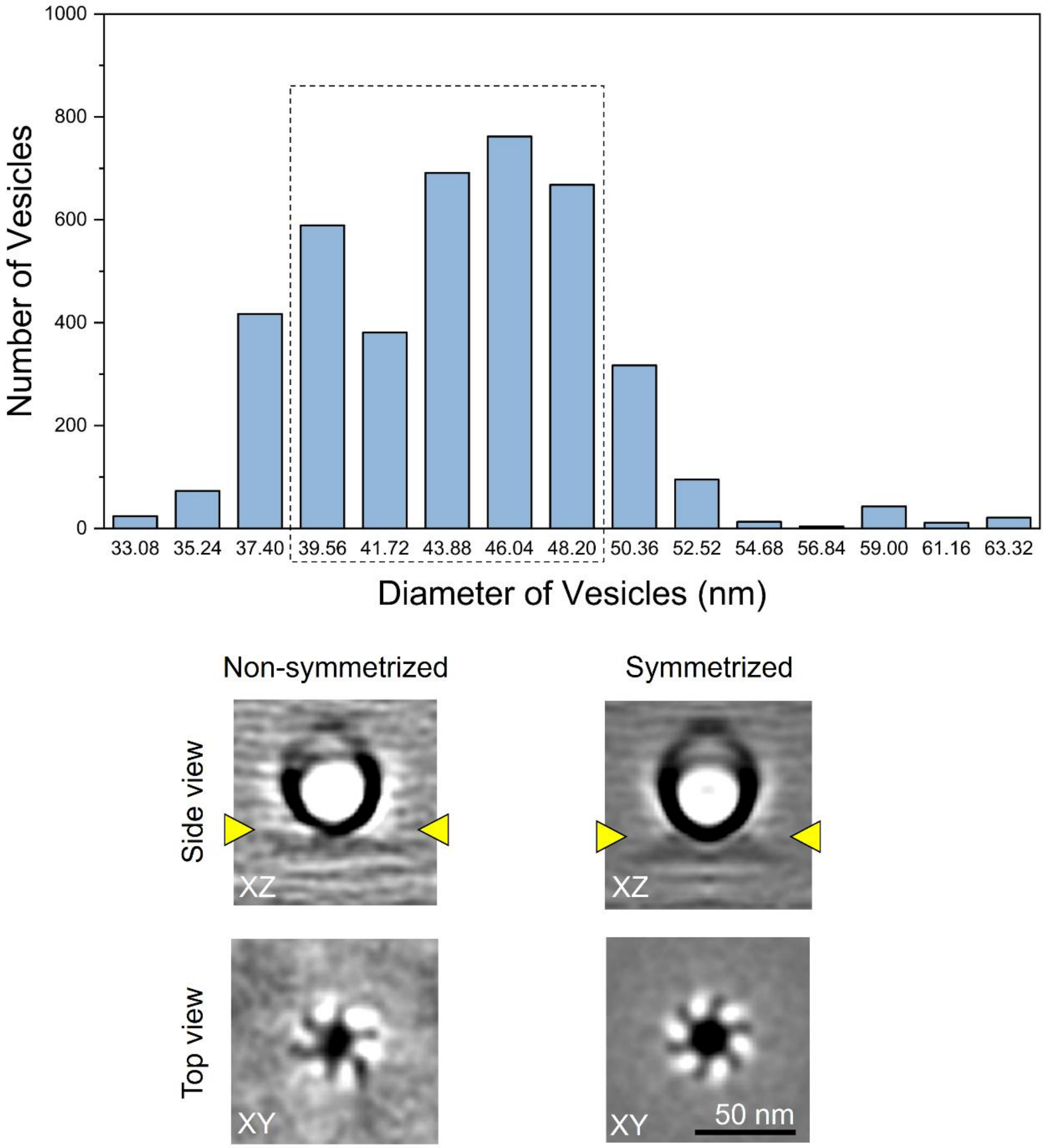

***Fig. S2**: Protein organization under 'top/bottom-view' docked vesicles.*

Distribution of 'top/bottom-view' docked vesicles as a function of synaptic vesicle (SV) diameter. The thickness of the bilayer was assumed to be 5 nm for all the calculations. Top/bottom-view vesicles (~5000 bin4 sub-tomograms with 1 pixel = 2.16 nm) were used for the analysis. SVs with a diameter of 43.88 ± 5.32 nm were pooled together to generate a homogeneous dataset for sub-tomogram averaging. The 3D reconstruction of the observed protein density, following several rounds of alignment and classification revealed a hexameric arrangement of protein densities at the docking interface either with or without an imposed six-fold rotational symmetry (bottom panel). The yellow arrow heads represent the vertical position in the SV-PrM interface (in the XZ slice or side view) at which the XY plane (or top view) is rendered.

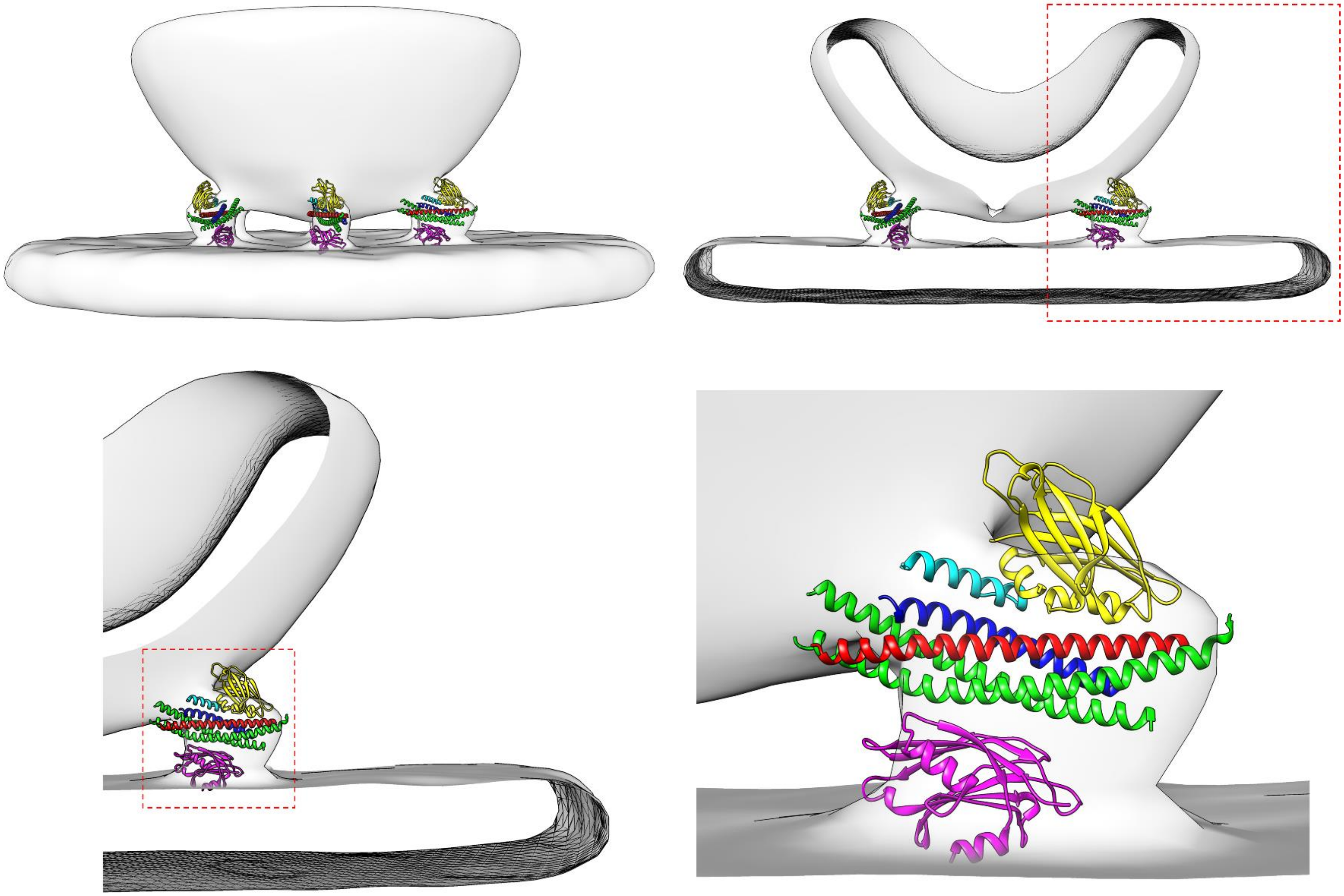

Fig. S3*: Cryo-ET 3D reconstruction of protein organization at SV-PrM interface.*

Rigid body fitting of crystal structures of constituent proteins into the densities connecting the SV to the PrM. Given the relatively low resolution, it is not possible to rigorously assign specific proteins to observed densities in the 3D map, but each of the pronounced density observed between the SV and PrM could accommodate exocytic module consisting of synaptic SNARE proteins (SNAP-25 – green, Syntaxin-1A – red, VAMP2 – blue); Complexin (cyan), two Synaptotagmin C2B molecules – primary (magenta) and tripartite (yellow). Note: Fitting into the six-fold rotationally symmetrized cryo-ET map at threshold level σ = 0.5 is shown and Syt1 C2A domains are omitted for clarity. The crystal structure (SNARE-Syt1-Complexin; PDB code 5W5C) was manually fitted into the cryo-ET map using USCF Chimera software.

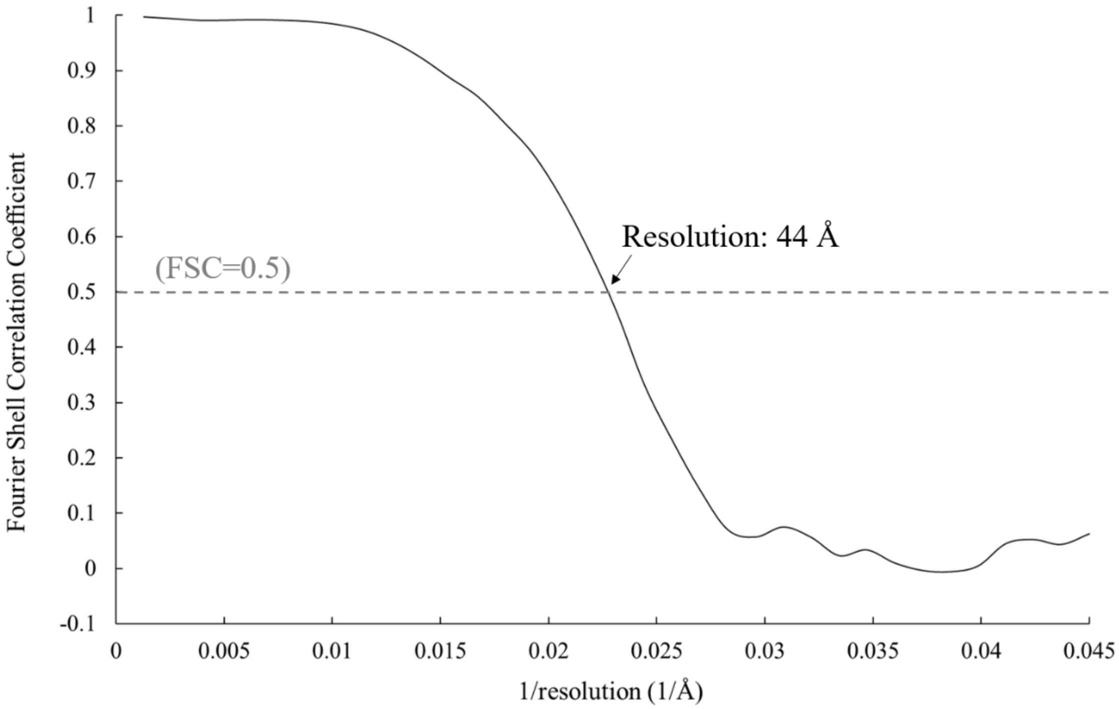

Fig. S4*: Fourier Shell Correlation (FSC) between the two half maps of the final 3D reconstruction with a soft mask applied on the protein densities connecting the SV and PrM.*

Supporting Movie Legends

Movie S1*: Representative tomogram of the synapse of a primary hippocampal neurons grown directly on the EM grids. Scale bar = 200 nm.*

Movie S2*: Representative tomogram shows the distribution of synaptic vesicles, at various stages of docking, in the neuronal synapse. Scale bar = 100 nm.*

Movie S3*: Animation of the 3D segmentation rendering of the reconstructed tomogram shows the detailed ultrastructure of a neuronal synapse. Scale bar = 100 nm.*
